# Infertilité primaire révélant un syndrome de Rokitansky: à propos d’un cas

**DOI:** 10.11604/pamj.2025.52.113.49366

**Published:** 2025-11-14

**Authors:** Raddaoui Awatef, Zouari Marouan, Raddaoui Yosra, Chibani Mounir

**Affiliations:** 1 Service de Gynécologie-Obstétrique, Hôpital Militaire Principal d'Instruction de Tunis, Tunis, Tunisie

**Keywords:** Aménorrhée, infertilité, plasie utéro-vaginale, cœlioscopie, cas clinique, Primary amenorrhea, infertility, utero-vaginal aplasia, laparoscopy, case report

## Abstract

Le syndrome de Mayer-Rokitansky-Küster-Hauser (MRKH) est une malformation congénitale rare caractérisée par une aplasie utérine associée à une agénésie des deux tiers supérieurs du vagin chez des femmes phénotypiquement féminines et présentant un caryotype 46,XX. Nous rapportons le cas d'une patiente âgée de 22 ans, consultante pour infertilité primaire. L'examen clinique retrouvait une vulve d'aspect normal, avec une cavité vaginale réduite à une cupule superficielle. L'échographie pelvienne et l'imagerie par résonance magnétique (IRM) ont objectivé une agénésie complète de l'utérus et des deux tiers supérieurs du vagin, avec des ovaires normaux. Le bilan hormonal montrait une fonction ovarienne conservée, et le caryotype était 46,XX. Une cœlioscopie diagnostique a permis de confirmer le diagnostic du syndrome de MRKH de type I. Ce cas illustre l'importance d'évoquer ce diagnostic devant toute aménorrhée primaire, même révélée tardivement dans un contexte d'infertilité.

## Abstract (en)

Mayer-Rokitansky-Küster-Hauser (MRKH) syndrome is a rare congenital malformation characterised by uterine agenesis and absence of the upper two-thirds of the vagina in phenotypically normal females with a 46,XX karyotype. We report the case of a 22-year-old woman presenting with primary infertility. Clinical examination revealed normal external genitalia with a shallow vaginal dimple. Pelvic ultrasound and MRI showed complete agenesis of the uterus and upper vagina, with normal ovaries. Hormonal evaluation confirmed preserved ovarian function, and karyotyping revealed a normal 46,XX profile. Diagnostic laparoscopy confirmed the diagnosis of type I MRKH syndrome. This case highlights the importance of considering this diagnosis in any woman with primary amenorrhea, even when revealed late in the context of infertility.

## Introduction

Le syndrome de MRKH, également appelé syndrome de Rokitansky ou aplasie utéro-vaginale, est une malformation congénitale rare touchant environ une femme sur 4500. Il se caractérise par une aplasie de l'utérus et des deux tiers supérieurs du vagin, chez des patientes présentant un phénotype féminin normal et un caryotype 46,XX [1]. Le diagnostic est généralement évoqué devant une aménorrhée primaire chez des adolescentes âgées de plus de 15 ans, présentant une croissance staturo-pondérale normale et des caractères sexuels secondaires bien développés [2]. Si la découverte est le plus souvent précoce, certains cas peuvent être diagnostiqués plus tardivement, notamment dans un contexte d'infertilité primaire. Nous rapportons l'observation d'une patiente chez qui le syndrome de MRKH a été découvert à l'occasion d'un bilan d'infertilité. La patiente a consulté à l'âge de 22 ans pour infertilité primaire évoluant depuis deux ans. L'anamnèse a révélé une aménorrhée primaire. Les explorations cliniques et radiologiques réalisées la même année ont permis de poser le diagnostic de syndrome de MRKH type I. Une prise en charge psychologique a été instaurée et la patiente a été informée des options thérapeutiques disponibles.

## Patient et observation

**Information de la patiente:** il s'agit d'une patiente âgée de 22 ans, de groupe sanguin O Rhésus négatif, sans antécédents médicaux ou chirurgicaux notables. Elle s'est présentée en consultation externe de gynécologie-obstétrique de l'Hôpital Militaire Principal d'Instruction de Tunis pour bilan d'une infertilité primaire évoluant depuis 2 ans et 2 mois. À l'interrogatoire approfondi, la patiente rapportait n'avoir jamais eu de règles.

**Résultats cliniques:** l'examen général retrouvait une patiente consciente, avec un score de Glasgow à 15/15 et un abdomen sans particularités. Les seins étaient bien développés et les caractères sexuels secondaires conformes au phénotype féminin. L'examen gynécologique montrait une vulve d'aspect normal et un orifice vaginal présent, mais une cavité vaginale réduite à une cupule de quelques centimètres de profondeur, correspondant au stade V de la classification de Tanner et Whitehouse.

**Figure 1:**
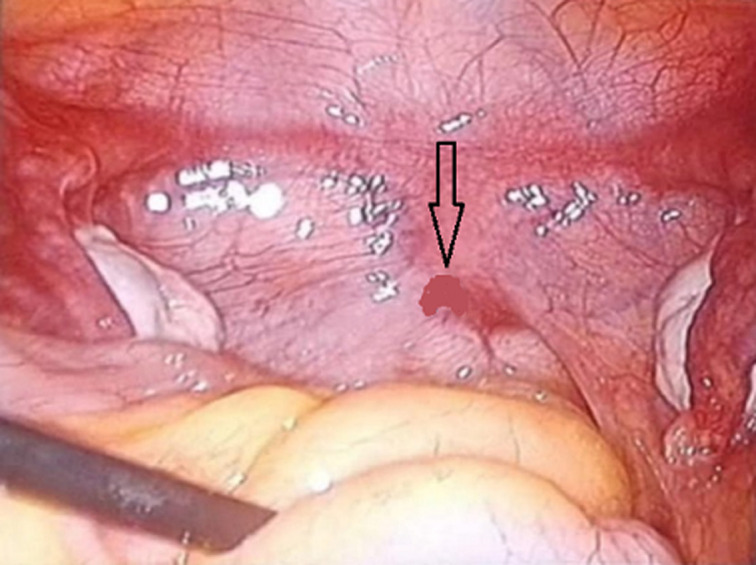
vue laparoscopique montrant l'absence d'utérus et la présence d'ovaires normaux, confirmant un syndrome de Mayer-Rokitansky-Küster-Hauser de type I

**Démarche diagnostique:** l'échographie pelvienne objectivait deux ovaires de taille normale, à aspect folliculaire, avec absence d'utérus. Le bilan hormonal confirmait une fonction ovarienne normale (œstradiol: 110pg/ml; FSH: 5,8mUI/ml; LH: 4,2mUI/ml; testostérone normale). Le karyotype était 46,XX. Une IRM abdomino-pelvienne a montré une agénésie complète de l'utérus et des deux tiers supérieurs du vagin, avec des ovaires normaux et des reins de localisation et d'aspect normaux. Une cœlioscopie diagnostique, réalisée en complément, a confirmé le diagnostic de syndrome de MRKH de type I (Figure 1).

**Intervention thérapeutique et suivi:** la patiente a bénéficié d'un soutien psychologique. Une prise en charge chirurgicale visant la création d'un néovagin pourra être envisagée secondairement afin d'assurer une vie sexuelle normale. La question de la fertilité a été abordée: la gestation étant impossible en l'absence d'utérus, la patiente a été informée des perspectives limitées, incluant la transplantation utérine encore expérimentale et non disponible localement.

**Perspective du patient:** la patiente a exprimé sa volonté de partager son expérience afin de contribuer à une meilleure compréhension du syndrome de Rokitansky dans le contexte de l'infertilité primaire. Elle espère que la publication de ce cas pourra sensibiliser d'autres femmes confrontées à cette situation et aider les cliniciens dans la prise en charge.

**Consentement éclairé de la patiente:** il a été obtenu pour la publication de ce cas et de toutes les données cliniques associées, dans le respect de l'anonymat.

## Discussion

Le syndrome de MRKH est défini par une aplasie congénitale de l'utérus et des deux tiers supérieurs du vagin, chez des patientes présentant un développement pubertaire normal et un caryotype 46,XX [1]. Il peut se présenter sous deux formes: type I: forme isolée, limitée aux anomalies utéro-vaginales; type II: forme associée à d'autres malformations, notamment rénales, musculo-squelettiques, auditives ou cardiaques [2]. Sur le plan embryologique, l'aplasie utéro-vaginale correspond à un défaut de développement ou de migration des canaux de Müller vers le sinus urogénital. L'étiologie exacte reste mal élucidée, mais une origine polygénique et multifactorielle est la plus probable [1]. Cliniquement, le syndrome est le plus souvent diagnostiqué à l'adolescence, devant une aménorrhée primaire chez une jeune fille ayant un développement mammaire et pileux normal [2]. Dans notre observation, la particularité réside dans la découverte tardive, lors d'un bilan d'infertilité primaire.

Le bilan diagnostique repose sur l'examen clinique, l'exploration hormonale, le caryotype et surtout l'imagerie. L'échographie pelvienne peut révéler l'absence d'utérus, mais l'IRM constitue l'examen de référence, permettant de confirmer l'agénésie utérine et vaginale et de rechercher d'éventuelles anomalies associées [3]. La cœlioscopie peut être indiquée pour compléter le bilan anatomique et envisager une prise en charge chirurgicale [4]. Le traitement vise principalement la création d'un néovagin fonctionnel, afin d'améliorer la qualité de vie sexuelle. Les techniques non chirurgicales, comme la méthode de Frank, reposent sur une dilatation progressive de la cupule vaginale à l'aide de bougies ou de rapports sexuels réguliers. Les techniques chirurgicales comprennent la méthode de Vecchietti ou la création d'un néovagin à partir d'un segment intestinal [5].

Sur le plan de la fertilité, l'absence d'utérus rend la gestation impossible, sauf dans le cadre de la transplantation utérine, encore limitée à quelques centres spécialisés [6]. Le recours à une gestation pour autrui est une alternative dans certains pays, mais reste interdit dans de nombreuses législations.

## Conclusion

Le syndrome de MRKH est une cause rare mais majeure d'aménorrhée primaire et d'infertilité féminine. Son diagnostic repose sur l'association d'un examen clinique soigneux, d'un bilan hormonal et génétique et d'une imagerie pelvienne de haute résolution. La prise en charge, multidisciplinaire, vise avant tout à restaurer une sexualité satisfaisante grâce à la création d'un néovagin fonctionnel. La question de la fertilité demeure un défi, et les options actuelles restent limitées, avec la transplantation utérine comme seule perspective biologique.

## Remerciements

Les auteurs expriment leur gratitude à l'équipe du Service de Gynécologie-Obstétrique de l'Hôpital Militaire Principal d'Instruction de Tunis pour leur collaboration dans la prise en charge de la patiente. Ils remercient également la patiente pour sa confiance et son accord pour la publication de ce cas.
